# Impact of the astronomical lunar 18.6-yr tidal cycle on El-Niño and Southern Oscillation

**DOI:** 10.1038/s41598-018-33526-4

**Published:** 2018-10-12

**Authors:** Ichiro Yasuda

**Affiliations:** Atmosphere and Ocean Research Institute, The University of Tokyo, Kashiwanoha 5-1-5, Kashiwa, Chiba, 277-8564 Japan

## Abstract

Even though El-Niño and Southern Oscillation (ENSO) has a tremendous impact on global climate and society, its long-term forecast remains difficult. In this study, we discovered a statistically significant relationship between ENSO timing and the 18.6-year period lunar tidal cycle in the mature-phase (December–February) ENSO time-series during 1867–2015 and extending back to 1706 with proxy data. It was found that El-Niño tended to occur in the 1st, 10th, and 13th years after the maximum diurnal tide in the 18.6-yr cycle, and La-Niña tended to occur in the 3rd, 12th, and 16th years. These tendencies were also confirmed by corresponding sea-surface temperature (SST) and sea-level pressure (SLP) distributions; particularly Pacific SST and SLP spatial patterns in the third La-Niña and the tenth El-Niño year well resemble those of Pacific Decadal Oscillation (PDO). These findings contribute to understanding and forecasting long-term ENSO variability.

## Introduction

**Figure 1:**
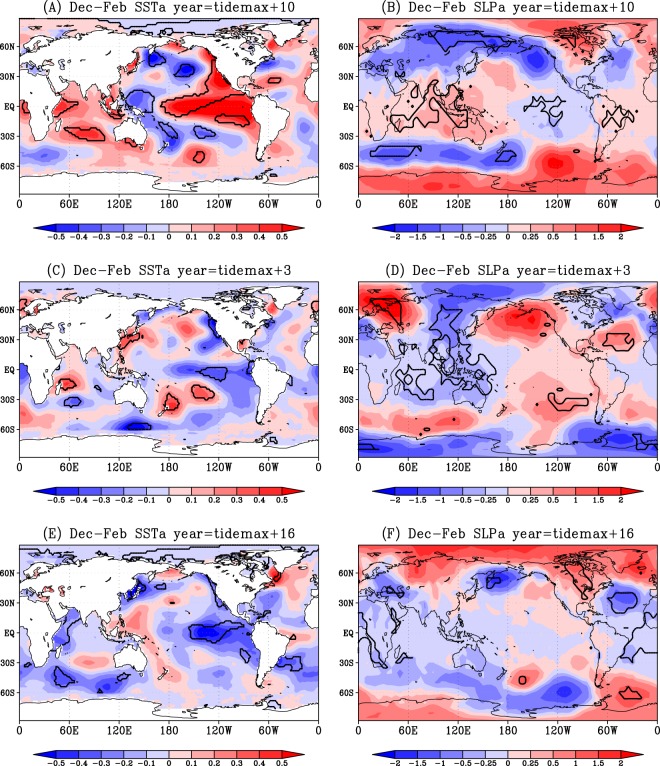
Composite of December-February SST (in °C, left panels) and SLP (in hPa, right panels) anomaly distributions in the 10th (El-Niño) and 3rd and 16th (La-Niña) tide years after the maximum diurnal tide. The areas surrounded by the thick black contours indicate the 95% confidence mean.

El-Niño^1^ is a phenomenon that sea-surface temperature (SST) in the equatorial Pacific east of 180° is warmer than usual (Fig. 1A), whereas during La-Niña, the SST is colder than usual (Fig. 1C). Coupled with this variability in SST, fluctuation in sea-level pressure (SLP) anomaly between east and west develops in the tropical Pacific (Fig. 1B,D). This is known as Southern Oscillation. These coupled air-sea phenomena are often referred to as El-Niño and Southern Oscillation (ENSO).

ENSO impacts global climate^2^ through atmospheric teleconnection^3,4^. Hence, ENSO has been studied intensively, and several theories^5–8^ of ENSO have been proposed. Seasonal ENSO forecast has been successfully performed on the basis of ENSO persistence: June-August anomaly persists and amplifies until the following winter in the northern hemisphere (February to March). However, the spring season that follows has almost no correlation with the previous winter (referred to as “spring barrier”)^9,10^, making long-term ENSO forecasting (beyond 1 year) difficult.

Finding the relationship between ENSO and external forcings with known period and phase would contribute to the long-term forecast of ENSO. One of possible candidates for this is the 18.6-year period moon tidal cycle^11^ (henceforth, 18.6-yr cycle). The bi-decadal component of Pacific Decadal Oscillation^12^ (the first principal component of SST variability north of 20°N in the North Pacific, henceforth PDO) is known to be related to the 18.6-yr cycle^13,14^. Since the SST and SLP pattern of PDO is similar to ENSO^12^, PDO is also referred to as ENSO-like variability which affects global mean surface temperature as “hiatus”^15^ (global warming slowdown). ENSO could, thus, be related to the 18.6-yr cycle.

The 18.6-yr cycle is caused by the oscillation of the orbital surface of moon around the earth: the surface inclines 23.4° to the equatorial surface of earth on average and this inclination oscillates with the period of 18.613 years with the amplitude of 5.2°^11^. This 18.6-yr cycle causes the long-term modulation of oceanic tides where amplitudes of diurnal (semi-diurnal) components of K_1_ and O_1_ (M_2_) tides modulate by 11% and 19% (4%) respectively, and the amplitude modulation of the semi-diurnal M_2_ tide is out-of-phase from the diurnal (K_1_ and O_1_) tides^11^. This tidal modulation causes variations in oceanic vertical mixing variability, and could influence the oceanic and eventually climatic variability^11,13^.

An observational study has suggested the relationship between the 18.6-yr cycle and the timing of ENSO; stronger-than-usual El Niño events tend to occur during the periods of weak diurnal tide and low inclination of orbital surface of moon^16^. This is based on the information that NINO3.4 (5°S-5°N and 170°W-120°W) SST^17^ anomaly in the central-eastern equatorial Pacific (index for monitoring ENSO) is positive (Fig. S1A in Supplementary Information A), and the mean Southern Oscillation Index (SOI^17^: difference in normalized SLP, Tahiti (17°38′S, 149°27′W) minus Darwin (12°27′S, 130°50′E)) is negative (Fig. S1B) during the 5 (~18.6/4)-year period at around the minimum diurnal tide. However, when the 95% (90%) confidence interval is evaluated by $1.96\,(1.645)\times \sigma /\sqrt{{N}_{c}},$ where *σ* is the standard deviation and *N*_c_ is the degree of freedom, the means were not found to be significant. In the previous study, *N*_c_ were set at number of months^16^. However, monthly data cannot be considered independent due to the seasonal persistence of ENSO, and, thus, number of months is not appropriate for the degrees of freedom, and number of years should be used.

## Data and Methods

A composite analysis was performed in each tide year for the mature phase (December–February: DJF) ENSO (NINO3.4, SOI and NINO1 + 2: 0–10°S 90°W-80°W) time-series data^17^ of 1867–2015 (148 years). A 310-year-long (mostly doubled) DJF SOI time-series extending back to 1706 was also used by adding the reconstructed proxy data^18^ using tree-ring chronology for 1706–1866. The proxy data was adjusted (multiplied by 0.3) to the instrumental SOI^17^ in the overlapping period of 1867–1977 and then subtracted by the 310-year mean (−0.17). The composite analysis was also applied to global SST^19^ and SLP^20^ data.

The 0 year was set at the maximum (minimum) diurnal (semi-diurnal) tide. In the 9th–10th year, diurnal tide took the minima. El-Niño (La-Niña) tends to occur when the mean in a certain tide year is positive (negative) beyond the confidence interval of the mean. 95% (90%) confidence interval of the mean was evaluated using the formula $1.96\,(1.654)\times {\rm{\sigma }}/\sqrt{{N}_{c}},$ where *σ* is the standard deviation and the degrees of freedom *N*_*c*_ ≈ *N*/18.6 is conservatively defined as the tidal cycle number where *N* is the total number of data points (year).

The tide year after the maximum diurnal tide in the 18.6-yr cycle, *Y*_Tide_ (=*Y* − *Y*_min_: *Y* is the year of the data and *Y*_min_ is the year of nearest minimum diurnal tide satisfying *Y* ≧ *Y*_min_) was evaluated as follows. Since the year of 1969.25 had one of the maximum diurnal tides and the accurate period of the cycle is 18.613 years, the years *Y*_min_ at the maximum diurnal tide were calculated by rounding down to the decimal of (1969.25 + 18.613 × *i* + *Y*_a_), where *i* = 0, ±1, ±2, … and *Y*_a_ = (7 − *m*)/12 with *m* (=1, …, 12) for the 1st day of each month and *m* (=1.5, 2.5, …, 12.5) for the day 15th of each month. *Y*_a_ = 0.458 (=(7–1.5)/12) was used for the DJF mean time-series in the present study. The addition of *Y*_a_ is necessary to accurately compute the nearest years of the maximum tide for a certain month of the data because the interannual nature of ENSO variability influences on the analysis and the tide year depends on month and day of the data used. For example, *Y*_min_ near the maximum of 1969.25 is 1968 for the data from 2 October to 31 December, and 1969 for the data on 1 January to 1 October.

Even though some of the means were significantly different from zero, it is too early to be concluded that ENSO is related to the 18.6-yr tidal cycle. Even if ENSO occurred randomly, such random time-series with limited data length could yield some significant means. Hence, to estimate the appearance probability of such significant means from random time-series (so-called False Discovery Rate, FDR), Monte-Carlo simulations were performed using 100,000 pseudo time-series with the same 1-year lag auto-correlation (*r* ~ ±0.04) (i.e., red spectra), data length, standard deviation and initial conditions as those of the ENSO time-series. The time-series were generated using the formulation $y(t)=r\times y\,(t-1)+{\sigma }_{W}\times \varepsilon (t)$ where *y*(*t*) is the time series in the year *t*, *r* is the 1-year lag auto-correlation coefficient of the ENSO time-series, ${\sigma }_{W}(={\sigma }_{R}\sqrt{1-{r}^{2}\,})$ is the standard deviation of the normalized white noise *ε*(*t*), and *σ*_*R*_ the standard deviation of the ENSO time-series. Estimates of FDR using an alternative standard method^21^, which may be too simple to be applied to this study, were described in Supplementary Information B and Table S1.

## Results

**Figure 2:**
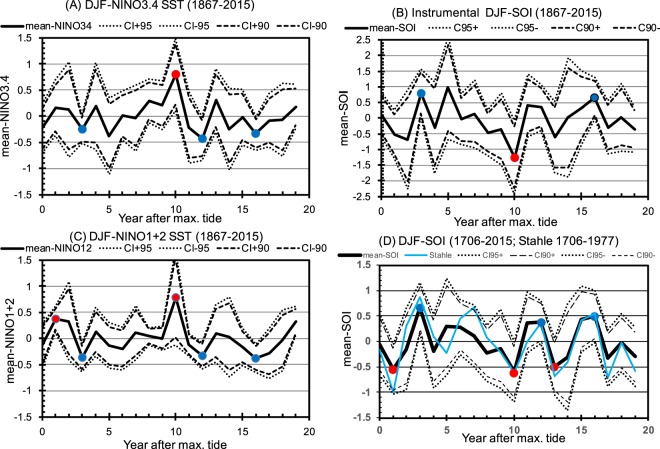
Mean and confidence intervals of December–February (**A**) NINO3.4 SST (°C), (**B**) SOI, (**C**) NINO1 + 2 SST (°C) and (**D**) extended SOI with reconstructed proxy data in each year after the maximum diurnal tide of the 18.6-yr cycle. The thick solid, broken and dotted lines denote means, 90% and 95% confidence intervals (CI). Red and blue dots denote the significant occurrence of El-Niño and La-Niña, respectively, in which the means ± CI are deviated from zero. The blue curve in (**D**) denotes the mean-SOI from the original Stahle-proxy data during 1706–1977.

A significantly (at 95% level) positive (negative) NINO3.4 SST, suggesting El-Niño (La-Niña), occurred in the 10th (3rd, 12th and 16th) year after the maximum diurnal tide in the 18.6-yr cycle (Fig. 2A), and significantly (at 95% level) negative (positive) SOI occurred in the 10th (3rd and 16th) year (Fig. 2B). For another ENSO index of the NINO1 + 2 SST for the eastern south Pacific off Peru, positive (negative) anomaly occurred in the 1st and 10th (3rd, 12th and 16th) tide years (Fig. 2C; 90% significance for 10th tide year and 95% for other tide years). The 10th year for El-Niño and the 3rd and 16th year for La-Niña are common for those 3 ENSO indices for the 148 years and 8 cycles of the 18.6-yr period.

**Figure 3:**
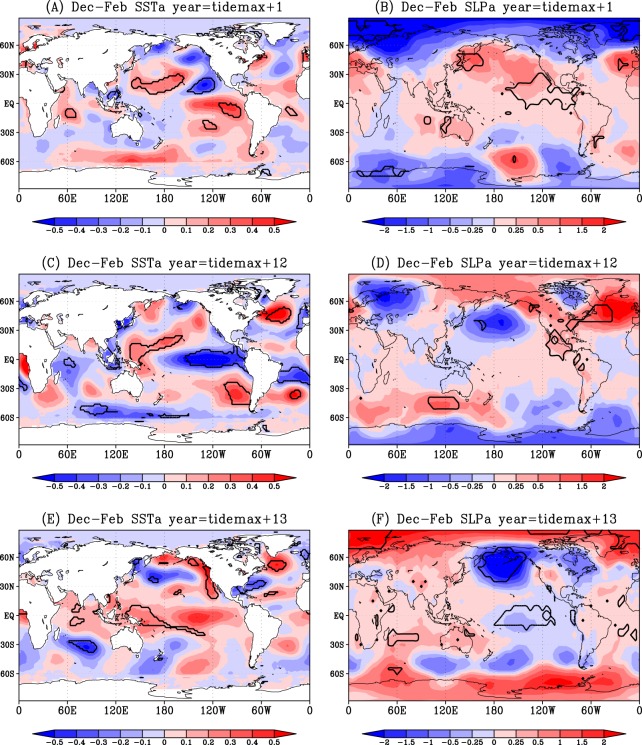
Same as Fig. 1 but for the anomaly distributions in the 1st and 13th (El-Niño) and 12th (La-Niña) years after the maximum diurnal tide.

Horizontal anomaly distributions of DJF SST^19^ during 1854–2015 and SLP^20^ during 1860–2015 in the common 3rd, 10th and 16th tide year (Fig. 1) confirm the ENSO tendency as indicated by the ENSO indices (Fig. 2). SST and SLP anomaly distributions in the tropical Pacific in the 1st (Fig. 3A,B) and 12th (Fig. 3C,D) tide year also confirm the ENSO tendency from NINO1 + 2 SST (Fig. 2C for the 1st and 12th year) and NINO3.4 SST (Fig. 2A for the 12th year).

These significantly negative (positive) means of SOI suggesting El-Niño (La-Niña) tendency, were also found during the 310-year-long 1706–2015 DJF SOI time-series (Fig. 2D). Significantly negative (positive) SOI occurred in the 1st, 10th and 13th (3rd, 12th and 16th) year after the maximum diurnal tide (Fig. 2D). The horizontal SST and SLP patterns in the 13th tide year (Fig. 3E,F) confirms the El-Niño tendency.

Statistical significance of the present composite analysis was further evaluated as the random processes may generate significant means for relatively short time-series. Monte-Carlo simulations showed that 95%-significant means in ≧1, 2, 3, 4, 5, and 6 tide years appear at the probabilities of 86, 57, 29, 11, 3.4, and 0.8%, respectively, from 148-year-long random pseudo time-series (Table 1A). The 3, 4, and 5 (4 at 95% significance level and 1 at 90%) significant means corresponding to the SOI, NINO3.4-SST and NINO1 + 2-SST, respectively appear at the probabilities of 29%, 11%, and 8%, respectively, from pseudo random time-series. Hence, it cannot be concluded that the 18.6-yr cycle and ENSO relations in the short (145 yr) time-series are significant at 95% level.

**Table 1:** Appearance probability (in %) of significant means for tide-year number (≧1, 2, …) from (A) 148 and (B) 310-year-long pseudo random time-series with the same 1-year lag correlation and initial condition as the observed SOI. 5 (4) 95% significant means appear at less than 5% probability from 145 (310) year time series. (C) Appearance probability of the case where tide years with significant means are common between in 148 and 310-yr time series. For example, 3 (4) 95 (90)% significant means at the same tide year in the 145 and 310-yr time-series appear at 1.4%.

Number of years with significant means	(A) SOI (148-yr)		(B) SOI (310-yr)		(C) Same tide years of significant means between 145 and 310-yr SOI	
	Confidence Interval CI95%	CI90%	CI95%	CI90%	CI95%	CI90%
≧1	86%	95	72	89	40.1	62.5
≧2	57	80	36	65	9.3	25.1
≧3	29	55	13	37	1.4	7
≧4	11	32	3.3	16	0.14	1.4
≧5	3.4	15	0.7	5.9	0.015	0.22
≧6	0.8	5.4	0.11	1.7	0	0.02

In contrast, the four 95% and two 90% significant means in the 310-year-long SOI (Fig. 2D) appear only at 0.9% probability from random time-series, leading to the conclusion that ENSO is not completely random and is related to the 18.6-yr cycle at 99% confidence level. Even when the 310-year mean (−0.17) was not subtracted from the time-series, El-Niño (La-Niña) tendency in the 1st, 10th and 13th (3rd) tide year was still found to be significant at 95%, which appeared only at 3.3% probability (Table 1B). The probability of the four 95% significant means being common (occur in the same tide year) between in the short 145-year (Fig. 2A,C) and the long 310-year (Fig. 2D) time-series is 0.14% (Table 1C), further supporting the robustness of the relation between ENSO and the 18.6-yr cycle.

## Discussion

Although physical mechanisms which explain the relation between ENSO and 18.6-yr cycle are beyond the focus of the present study and relied on future studies, possible mechanisms are discussed here. A temporal scale difference exists between interannual ENSO and bi-decadal 18.6-yr cycle. 5 × *f*_18.6_ frequency (five times 18.6-yr cycle frequency) variability (with the period of 3.7 (=18.6/5) years) which is particularly depicted in the original proxy Stahle-SOI timeseries (blue curve in Fig. 2D has 5 maxima and 5 minima) may be generated through nonlinear dynamical processes and be resonated with equatorial waves^5–8^ which are bounded by eastern and western coasts to generate the relation of interannual-scale ENSO and the 18.6-yr cycle. These processes need to be examined in future studies.

The second discussion is the location and mechanism of the 18.6-yr period forcing. One possible location could be the remote forcing from mid-latitudes, and the other could be the direct forcing in the tropical Pacific. The remote forcing was previously examined with an air-sea coupled climate model^22^ with locally enhanced vertical mixing and its 18.6-yr period variability around the Kuril Straits which border the North Pacific and the Okhotsk Sea. Anomalous stratification and currents generated around the Kuril Straits propagate along the western boundary as coastally trapped waves to change the equatorial regions. This generates the 18.6-yr period ENSO-like (PDO) variability with its peak in the 6th tide year for La-Niña-like (negative) PDO and 15th year for El-Niño-like (positive) PDO. The relationship between this model-PDO and 18.6-yr cycle is similar to the previous observational one^14^. The enhanced tide-induced vertical mixing assumed in the climate model around the Kuril Straits was confirmed with direct turbulence observations^23,24^, and the evidence of 18.6-yr water-mass variability was reported in the subarctic North Pacific and marginal seas^25–29^. Another climate model experiments^30^ with globally estimated distribution of tide-induced vertical mixing and its 18.6-yr modulation also confirmed that the ENSO-like PDO variability occurs similarly to the earlier model^22^ and the observation^14^, but the model exhibits weaker response in the equatorial and tropical Pacific. This might be because the direct forcing of the 18.6-yr cycle was underestimated in the model.

**Figure 4:**
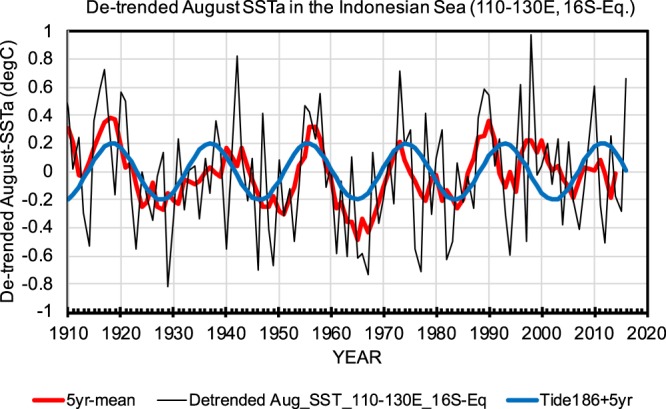
De-trended (during 1910–2015) August-SST anomaly (in °C) time-series averaged in 110–130°E and 16°S-0° in the Indonesian Sea (black solid curve), its 5-year running means (red) and 5-year lagged 18.6-year cycle (blue).

Evidence of the direct forcing of the 18.6-yr cycle in the equatorial Pacific was demonstrated in the de-trended (warming trend) August-SST in the Indonesian seas during 1910–2015 (Fig. 4). August SST was used because ENSO developed during August before strong air-sea interactions masked the influence of tidal mixing variability. In the Indonesian seas, where semi-diurnal M_2_ tide is dominant and takes the maxima (minima) in the 9th (0th) tide year, negative (positive) SST caused by the enhanced (weakened) tide-induced mixing could lead to positive (negative) SLP anomaly and thus to El-Niño (La-Niña). The 5-year running mean SST (red curve in Fig. 4) followed the 18.6-yr cycle with the 1/4 phase (about 5 years) lag, except for the low-SST in the early 1990s which corresponded to the 1991-Pinatubo eruption after which El-Niño tended to occur^31^. This lag of several years is consistent with the La-Niña (El-Niño) -like PDO tendency in the 3rd–5th (10–13th) tide year. The reasons of this lag need to be examined in future studies.

The direct 18.6-yr period forcing in the Indonesian seas (Fig. 4) may explain the simultaneous occurrence of El-Niño (La-Niña) in the 10th (3rd) tide year and positive (negative) PDO in the tide years of 10–13th (3rd–5th)^14^. The response of atmospheric stationary Rossby waves to the SST anomaly in the tropical Pacific, including the Indonesian seas (Fig. 4), possibly extends to mid-latitude North Pacific through teleconnections^2^, which are consistent with the SST and SLP patterns of PDO^12^. This simultaneous occurrence of ENSO and PDO suggests that ENSO and PDO may strengthen each other in the 10th (3rd) tide year through longer 18.6-yr-time scale PDO may set the environment where ENSO is remarkable and El-Niño (La-Niña) enhances positive (negative) PDO.

The robust relation between ENSO and 18.6-yr cycle found in this study is critical to understand and forecast the long-term ENSO variability. The 10th tide year corresponds to the recent two big El-Niño events in 1997/1998 and 2015/2016 data for which is not included in this study. 2017/2018 La-Niña corresponding to the 12th tide year is also successfully predicted by the relation (see Supplementary Information C and Table S2 showing the year when El-Niño and La-Niña occurred for NINO3.4 and SOI). The 2015/2016 big El-Niño was also predicted on the basis of a correlation between PNA (Pacific North American) SLP pattern and 18.6-yr cycle^25^. Underlying physical mechanisms of the relationship between ENSO and 18.6-yr cycle are required to be further explored by observing mixing processes and developing better climate models with refined tide-induced vertical mixing especially in mixing hot spots including the Indonesian seas.

## Electronic supplementary material

Supplementary Information

## Data Availability

All the data used in this paper is available.
