# Supplementary material for: Impact of the astronomical lunar 18.6-yr tidal cycle on El-Niño and Southern Oscillation
Source: Sci Rep. 2018 Oct 12;8:15206. doi: 10.1038/s41598-018-33526-4 (PMC6185980; doi:10.1038/s41598-018-33526-4)
Supplement: Supplementary file 1 — Supplementary Information [file 41598_2018_33526_MOESM1_ESM.pdf]

Supplementary Information,

Impact of the astronomical lunar 18.6-yr tidal cycle on El-Niño and Southern Oscillation

Sept. 8, 2018

Ichiro Yasuda

Atmosphere and Ocean Research Institute, The University of Tokyo

Kashiwanoha 5-1-5, Kashiwa, Chiba 277-8364, Japan

ichiro@aori.u-tokyo.ac.jp

A) Re-examination of the analysis of Cerveny and Shaffer (2001) with the appropriate degrees of freedom

The means in the quarter period of 18.6-yr cycle around the maximum diurnal tide are not significant (P=0.23) as shown in Fig. S1 when the degrees of freedom are set at the number of years instead of the number of months in Cerveny and Shaffer (2001) (CS01) because of the annual ENSO persistence. This indicates that the conclusion of CS01 that strong El-Niño tends to occur is not validated.

Figure S1: Mean (dots) and its 95% confidence interval (upper and lower bars) of December-February ENSO indices, (a) NINO3.4 SST (^{o}C) and (b) SOI for the four periods of the 18.6-yr cycle: at around maximum diurnal tide, from strong to weak diurnal tide, at around minimum diurnal tide, and from weak to strong diurnal tide. This figure shows the results of Cerveny and Shaffer (2001) are not significant because the degree of freedom is not properly defined.

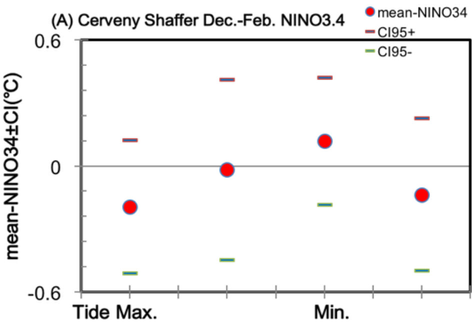

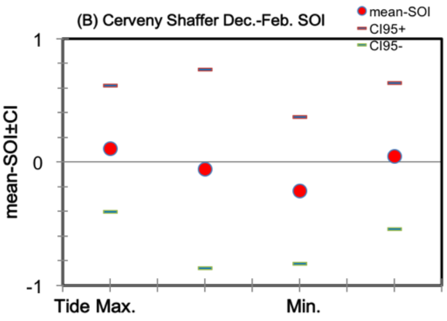

B) Estimate of 'False Discovery Rate[21] (FDR)' for the present 'multiple comparison problem'

The analysis using the Monte-Carlo simulations in the present study attempted to estimate how many trials reproduce the number of significant means from 100,000 trials with pseudo random time-series corresponding to the original ENSO data. The appearance probability in % is defined as the number of the reproducing trials divided by 1,000 as shown in Table 1. Since the appearance probability for the extended SOI time-series is less than 1%, it is concluded that the means are not from random processes at 99% confidence level, leading to the conclusion that ENSO is related to the 18.6-yr cycle. Alternative commonly-used approach for this kind of multiple comparison problem called 'False Discovery Rate' (FDR)[21] is here estimated.

FDR[21] is estimated as follows: 1) compute P-values of the means in 18 tide years (18 tests) from 0th to 17th tide year. 2) Compare the P-values with a guess-FDR $\times \frac{i}{18}$ $(i = 18,\ 17, \cdots, 1)$ from the largest to smallest P-values for the guess-FDR (for example, guess-FDR=0.05 meaning overall 5% false discovery rate), and if at least one P-value is less than the guess-FDR $\times \frac{i}{18}$, we know that FDR is less than the guess-FDR. 3) Seek the smallest FDR by repeating the 2)-procedure by changing the guess-FDR.

The false discovery rates (FDR) estimated are 0.24, 0.39, 0.1, and 0.15 for NINO3.4, SOI, NINO1+2, extended (310yr)-SOI respectively (Table S1). All the FDRs are not less than 0.05 (5%), although the Monte-Carlo simulation showed that extended SOI with the 6 significant means is at 99%-significance level. The smallest FDR is 0.1 for NINO1+2 with the 5 significant means. The FDRs are sensitive to the smallness of the P-values averaging the P-values of detected significant means (mean-P in Table S1) rather than the number of significant means (#-means in Table S1). This too-much sensitivity of the FDR[21] to the smallness of P-values could lead to the difference

between the FDR[21] and the one estimated using the Monte-Carlo simulations in this study.

Table S1: List of False Discovery Rate (Benjamini & Hchberg, 1995)[21] (FDR: 4th column) and FDR in this study from the Monte-Carlo simulation (Monte-Carlo: 5th column) for the time-series data (1st column), and corresponding figures and degree of freedom (DoF) are listed in the 2nd and 3rd column. The FDR[21] is sensitive to the mean P-value (mean P in the 7th column) averaging the P-values of detected significant means rather than the number of significant means (#-means in the 6th column).

Time-series	Figure	DoF	FDR	Monte-Carlo	#-means	mean P
NINO3.4	Fig. 2A	7-8	0.24	0.11	4	0.039
SOI	Fig. 2B	7-8	0.39	0.29	3	0.039
NINO1+2	Fig. 2C	7-8	0.1	0.08	5	0.014
extended-SOI	Fig. 2D	16	0.15	0.009	6	0.033

C) Time tables of El-Niño and La-Niña occurrence along the 18.6-yr tidal cycle from DJF NINO3.4-SST (Table S2A) and from extended DJF-SOI (Table S2B)

Table S2: Tables of El-Niño and La-Niña occurrence to check the tide years when El-Niño (1st, 10th and 13th) and La-Niña (3r, 12th and 16th) tends to occur in the (A) DJF-NINO3.4 SST (Fig. 2A) and (B) the extended DJF-SOI (Fig. 2D). Heavy and light orange and blue shadings denote the El-Niño and La-Niña with the absolute DJF-mean NINO3.4 SST (SOI) anomalies exceed 0.4 and 0.1 ºC (0.79 and 0.16) corresponding to 0.5 and 0.1 of the standard deviation. The year is for the month of January-February.

(A)

Tendency		EL-NINO	La-Nina	EL-NINO	La-Nina	EL-NINO	La-Nina
Tide Year	0	1	3	10	12	13	16
	1876	1877	1879	1886	1888	1889	1892
	1895	1896	1898	1905	1907	1908	1911
	1913	1914	1916	1923	1925	1926	1929
	1932	1933	1935	1942	1944	1945	1948
	1951	1952	1954	1961	1963	1964	1967
	1969	1970	1972	1979	1981	1982	1985
	1988	1989	1991	1998	2000	2001	2004
	2006	2007	2009	2016	2018	2019	2022
	2025	2026	2028	2035	2037	2038	2041
	2044	2045	2047	2054	2056	2057	2060
	2062	2063	2065	2072	2074	2075	2078
	2081	2082	2084	2091	2093	2094	2097
	2099	2100	2102	2109	2111	2112	2115
	2118	2119	2121	2128	2130	2131	2134
	2137	2138	2140	2147	2149	2150	2153
	2155	2156	2158	2165	2167	2168	2171

(B)

Tendency		EL-NINO	La-Nina	EL-NINO	La-Nina	EL-NINO	La-Nina
Tide Year	0	1	3	10	12	13	16
	1690						1706
	1709	1710	1712	1719	1721	1722	1725
	1727	1728	1730	1737	1739	1740	1743
	1746	1747	1749	1756	1758	1759	1762
	1764	1765	1767	1774	1776	1777	1780
	1783	1784	1786	1793	1795	1796	1799
	1802	1803	1805	1812	1814	1815	1818
	1820	1821	1823	1830	1832	1833	1836
	1839	1840	1842	1849	1851	1852	1855
	1858	1859	1861	1868	1870	1871	1874
	1876	1877	1879	1886	1888	1889	1892
	1895	1896	1898	1905	1907	1908	1911
	1913	1914	1916	1923	1925	1926	1929
	1932	1933	1935	1942	1944	1945	1948
	1951	1952	1954	1961	1963	1964	1967
	1969	1970	1972	1979	1981	1982	1985
	1988	1989	1991	1998	2000	2001	2004
	2006	2007	2009	2016	2018	2019	2022
	2025	2026	2028	2035	2037	2038	2041
	2044	2045	2047	2054	2056	2057	2060
